# Sensory Characteristics Contributing to Pleasantness of Oat Product Concepts by Finnish and Chinese Consumers

**DOI:** 10.3390/foods9091234

**Published:** 2020-09-04

**Authors:** Oskar Laaksonen, Xueying Ma, Eerika Pasanen, Peng Zhou, Baoru Yang, Kaisa M. Linderborg

**Affiliations:** 1 Food Chemistry and Food Development, University of Turku, FI-20014 Turku, Finland; oskar.laaksonen@utu.fi (O.L.); xueyma@utu.fi (X.M.); eerika.pasanen@utu.fi (E.P.); baoru.yang@utu.fi (B.Y.); 2 School of Food Science and Technology, Jiangnan University, Wuxi 214122, China; zhoupeng@jiangnan.edu.cn

**Keywords:** oat products, consumers, liking, Check-All-That-Apply, cross-cultural, China, Finland

## Abstract

Oats are increasingly popular among consumers and the food industry. While data exist on sensory characteristics of oats as such, previous studies focusing on the pleasantness of oats, and especially investigations of a wide range of oat products by European and Asian consumers, are scarce. An online questionnaire was organized in Finland (*n* = 381; 83.7% Finnish) focusing on the liking and familiarity of oat products, followed by sensory tests in Finland (*n* = 65 and *n* = 73) and China (*n* = 103) using the Check-All-That-Apply method and hedonic ratings. A questionnaire revealed that the Finnish consumers rated the pleasantness and familiarity of several oat product categories, such as breads and porridges, higher compared to participants of other ethnicities. Sensory tests showed both similarities, e.g., porridges were described as “natural”, “healthy” and “oat-like”, and differences between countries, e.g., sweet biscuits, were described as “crispy” and “hard” by Finnish consumers and “strange” and “musty” by Chinese consumers. Sweet products were unanimously preferred. The ethnicity had an important role affecting the rating of pleasantness and familiarity of oat product categories, whereas food neophobia and health interest status also had an influence. The proved healthiness of oats was a crucial factor affecting the choices of consumers and their acceptance in both countries.

## 1. Introduction

Foods made of oats are traditionally used in many European countries. The importance of oats is increasing globally due to the global need for a shift to a plant-based diet and public health concerns. Oats provide excellent nutritional value and have substantiated health benefits as oat beta-glucans lower plasma cholesterol levels and attenuate postprandial blood glucose rise, and oat fibers increase fecal bulk [1,2,3,4,5,6,7,8,9]. One of the most traditional oat dishes is porridge made from rolled oats. However, nowadays, a variety of oat products and fractions are available for both consumers and industry. In Finland, for example, the food use of oats is rapidly increasing [10]. Yet, globally, only about 10% of the oat crop is used for human consumption [11], while the majority of oats are used as fodder. The shift in use from the fodder to human food requires research on the sensory characteristics and usage, whereas the substantiated health effects may also influence the choice of consumers. Oats are typically utilized as whole grain. Whole grain foods are generally recommended by official nutritional recommendations in various countries. However, the use of whole grain cereals may have negative effect on the sensory quality perceived by consumers [12].

The original odor of raw oats is somewhat mild and heat treatment and further processing significantly affects the sensory quality and results in a typical “oat-like” odor [13]. The odor and flavor of heated oats have been described as “nutty”, “toasted”, “sweet” and “cereal-like” [12,14]. Oats have a high content of lipids which results in oats and oat products being susceptible to oxidation. Rancidity in oats and oat products derives from both endogenous enzyme activity as well as processing and storage [14]. Rancid odors and bitter off-flavors produced during oxidation may result in the limited consumption of oat products [14]. The oxidation of lipids and the use of oats as whole grain may also result in increased bitterness by higher contents of oxidation products or phenolic compounds and their derivatives, respectively, in the oat products [12,15,16]. The origin of the oat may also have a role in the sensory properties of the oat product [17]. In a study by Hu et al. [17] the aroma of the *Avena nuda* L. oat flakes originating from China differed from the flakes of *Avena sativa* L. from USA, Canada or northern European countries Denmark or Sweden. Supplementation of fractions such as those rich in beta-glucan into beverage concepts can increase the oat-like flavor but also result in increased perceptions of rancidity [18]. In addition to odor and flavor characteristics, the appearance and texture have been noted to contribute to the liking and potential usage of oat products. Despite previous investigations and definition of the sensory characteristics of oats as such [10,12], there are only few previous studies focusing on the liking and acceptance of oats and oat products.

The cultural background of the consumers affects how food items are perceived and thus also the liking and usage of the products. A wide range of sensory studies have been conducted comparing different nationalities and countries and, especially, comparing consumers of the Western and Eastern locations of the world. Examples of categories studied include meat products [19], honeys [20] and ciders [21]. The cultural background may also affect the behavior and response style in the consumer tests. Examples of such differences include the use of scales [22,23] and creation of descriptors for foods in rapid sensory characterization tests [21]. However, sensory characteristics and their links to the liking of oats and oat-based products have been previously investigated mainly in areas with a Western diet, and with a narrow selection of products compared to those currently available in Scandinavia [24,25]. Global food brands also have a growing influence on Chinese consumers [26], and the global prevalence of various chronic diseases, such as obesity and cardiovascular diseases, further increases. Oats with several approved health claims in EU and USA, have global potential from a public health point of view. Whole grains in general are recommended in the Chinese Dietary Guidelines from 2016. There are indications that Chinese consumers may not be influenced by the nutritional labels or health claims on the food packages [27], as well as indications that because of the increasing health concerns, Chinese consumers appreciate safe and organic foods and are willing to pay more for them [28,29]. However, it has currently not been investigated how the consumers in Finland and China differ from one another in these perceptions. Additionally, the effect of the fact that oats are gluten free [30] on the consumers is currently unclear.

There is an increasing interest in exporting oat products from producing countries to growing markets in East Asia. Despite these growing interests, there are no previous studies investigating or comparing the acceptance of oat product concepts in a cross-cultural context in European countries, where the oat is traditionally consumed, and Eastern countries, such as China, where cereal grains are commonly used, but oats are not equally popular.

This study aimed to identify the key sensory characteristics contributing to the liking of various oat products by conducting an online questionnaire in Finland and sensory tests in Finland and China. The goal was to study the perceptions and acceptance of oats and oat products by Chinese and Finnish consumers. A special focus was laid on the impact of cultural background of the participants as well as the influence of perceptions to new foods [31] and a general interest in the healthiness of food [32]. The studied oat product concepts in the questionnaire and in the sensory tests were designed to include various traditional (such as breads and porridges), and more novel food items (such as oat-based dairy and meat replacements), aiming to cover a wide range of oat products present in the Finnish market. The Check-All-That-Apply (CATA) method [33] was chosen for the sensory tests to provide rapid characterization of the product concepts.

## 2. Materials and Methods

### 2.1. Participants in the Tests

Four studies were conducted (Table 1). Study 1 was conducted as an online questionnaire in Finland. It was mainly advertised in Turku, Finland, but available for volunteers from other Finnish cities as well. Studies 2 and 3 were sensory trials in Turku, Finland, whereas study 4 was conducted as a sensory trial in Wuxi, China. Turku and Wuxi are both cities with large universities attracting students from throughout the countries and globally and physically locate close to bigger cities (Shanghai is close to Wuxi; Helsinki and Stockholm are close to Turku). Study 1 was available for all volunteers in Finland, and the participation of foreigners, especially the Chinese living in Finland, was encouraged. Participants were mainly students and staff of the universities in Turku. The Chinese participants who took part in Study 1 were encouraged to also take part in studies 2 and 3. All participants in studies 2 and 3 completed the online questionnaire (study 1) prior to taking part in the sensory evaluations if they had not done so already. The participants of studies 2 and 3 were allowed, but not required, to take part in both of these tests in Finland. The participants in study 4 were students and staff members of Jiangnan University located in Wuxi, Jiangsu province, China. Overall, 66.9% of the answers were provided by Finnish participants, 25.6% by Chinese participants and 7.6% by other ethnicities.

### 2.2. Online Questionnaire

The questionnaire (study 1, Table 1) included demographic questions (such as gender, age, country of origin) and questions about general interest towards healthiness, awareness of the healthiness of food and general usage of products containing oats on the first page and ratings for familiarity and pleasantness of selected oat products on the second page. The interest and awareness were rated on seven-point scales (1 = not interested/aware; 7 = extremely interested/aware), whereas usage was rated on a seven-point scale with labels: 1 = 2–4 times a day; 2 = Once a day; 3 = 2–4 times a week; 4 = Once a week; 5 = 1–3 times a month; 6 = A few times a year; 7 = Never. Familiarity of the oat products was rated on five-point scales with verbal anchors (1 = “not familiar at all” to 5 = “extremely familiar”) and pleasantness on nine-point balanced hedonic scales (1 = dislike extremely to 9 = like extremely). On the third page of the questionnaire, the standardized Food Neophobia Scale (FNS) [31] and General Health Interest (GHI) scale [32] were rated on a seven-point scale. For both FNS and GHI, a respondent’s task was to indicate their extent of agreement (1, ‘‘disagree strongly”, to 7, ‘‘agree strongly”) with 10 or 8 statements, respectively, and the potential ranges of the FNS and GHI scale scores were between 10 and 70 or 8 and 56. At the end of the questionnaire, all participants volunteering to take part in studies 2 and 3 gave their contact details in order to link the questionnaire to the sensory evaluation. The questionnaire was created with the Webropol 2.0 survey software (Webropol Oy, Helsinki, Finland) in both Finnish and English.

### 2.3. Oat Samples

The samples in studies 2–4 were Finnish oat products from varying product categories (Table 2). The samples were commercially available products with oat as the main ingredient or based on a commercially available product custom made without additional flavoring ingredients. The samples were either obtained from the manufacturers or purchased from a local supermarket. Due to the large variety of oat products and product categories in the market, the sensory evaluations in Finland were divided into two separate parts: dry oat samples and moist oat samples. Bread samples were frozen (−18 °C) immediately after purchase for the test in Finland or immediately after arrival in China to ensure that all subjects received bread from the same batches. The samples tested in China were selected based on the results of the tests in Finland, as well as stability during transportation from Finland to China during the summer period, i.e., samples requiring cooled storage were not selected. Samples were prepared, if needed, based on the instructions on the product packages.

The samples in Turku were presented to the participants in glass cups (dry samples) or in disposable 50 mL transparent plastic cups with watch glasses as lids (moist samples). Only disposable cups were used in study 4 in China. All dry samples were served at ambient room temperature. The moist samples, i.e., the oat meat substitute and the porridges were served freshly prepared and warm (approx. +60 °C), and the drinkable samples (oat drink and oat powder) as well as the yoghurt-alternative products were served refrigerated (approx. +4 °C).

### 2.4. Sensory Evaluations in Finland and China

Three separate sensory characterization tests (Table 1) were carried out using the Check-All-That-Apply (CATA) method [33] and following nine-point balanced hedonic scales (categories from dislike extremely to like extremely) for appearance, odor, flavor and mouthfeel; seven-point scales for odor and flavor intensities; and a five-point scale for purchase interest of the oat samples shown in Table 2. The CATA attribute list consisted of 34 attributes presented on one page and in fixed order for all samples and panelists including odor, flavor, taste and textural attributes as well as more abstract descriptors (Table S1). Attributes on the CATA list were based on the existing literature related sensory properties of oat and other grains and their products (e.g., reviews by Heiniö et al. [12,14]) as well as the preliminary sensory tests by the research team (data not published).

The questionnaires for the sensory evaluations were created with Compusense five 5.6 software (Compusense Inc., Guelph, ON, Canada), and the data were collected in Finland in Finnish and English using the software, whereas printed paper forms in Chinese were used in China. Samples (8–10/study; shown in Table 2) were presented to the participants all at the same time in randomized order (all possible permutations design) with three-digit random codes on the sample cups. Participants were instructed to examine samples in the given order monadically and select all possible CATA attributes in the sample and finally rate pleasantness, intensities and the purchase interests on the scales before evaluating the next sample. Additionally, participants were instructed to drink water between each sample to rinse their mouths. Sensory tests were organized in controlled laboratory conditions in individual sensory booths at the University of Turku, Finland, or at the Jiangnan University, China.

### 2.5. Statistical Analysis

Hedonic liking data from the online questionnaire were analyzed using ANOVA, with oat product, country of origin (two classes: from Finland or other country), gender, GHI status and FNS status (Table 1) being the main effects tested along with two-way interactions. Agglomerative hierarchical cluster analysis was carried out on the FNS and GHI scores, and consumer liking data to identify potential clusters and ANOVA together with Tukey’s post hoc test were used to identify significant differences in liking between clusters. Principal component analyses (PCA) were used to study the correlations between CATA attributes (frequency by the panels) and samples (separate models created for studies 2–4 in Table 1, *n* = 8–10 and one model containing all samples from these studies, *n* = 27). Full cross validation was used to the select validated number of components or factors in the models. All ANOVA tests and cluster analyses were performed with IBM SPSS Statistics 25.0 (IBM Corporation, Armonk, NY), and multivariate analyses were carried out with Unscrambler X (version 10.5, Camo Software, Oslo, Norway).

## 3. Results

### 3.1. Participant Clusters Based on the Online Questionnaire

The FNS status of the participants in study 1 was used to classify participants as less or more neophobic based on their sum scores using cluster analysis models. Similarly, GHI status was used to classify participants as more or less interested in the healthiness of food. The sum ranges for “less” and “more” neophobic were 10–29 and 30–70, respectively, and for “less” and “more” health interested, 8–35 and 36–56, respectively. Cluster analysis with four class variables (gender, country of origin, FNS status and GHI status) identified five clusters of participants (Table 3). The largest cluster (1) consisted of Finnish female participants with a high interest towards the healthiness of food and low FNS status. Clusters 2 and 3 were less interested in the healthiness of food (i.e., low GHI score). The majority of the Finnish male participants were in cluster 3. Clusters 4 and 5 consisted of more food neophobic, yet health interested participants from Finland (cluster 4) and other countries (cluster 5).

Clusters differed in the rated interest in the healthiness of food, awareness of the healthiness of the food they are consuming and the general usage of products containing oats (Table 3). The clusters with a lower GHI score (2–3) were less interested in the healthiness of food, but equally aware of the healthiness of food compared to clusters with a higher GHI score. Cluster 5 with non-Finnish participants was less aware of the healthiness of food than cluster 4 with similar GHI and FNS status. The clusters with Finnish respondents (1–4) used oat products more often than cluster 5 which consisted of non-Finnish participants.

The most common reasons for the Finnish participants in the online questionnaire to select oat products were “flavor”, “healthy” and “rich in fiber” (Table S2). Among the non-Finnish participants, the “flavor” was not an equally often selected reason in comparison to the other two. In Study 4 in Wuxi, China, “healthy” and “flavor” were among the most often selected reasons, whereas “easy” was also among the top three reasons.

### 3.2. Sensory Descriptors of Oat Products

**Figure 1:**
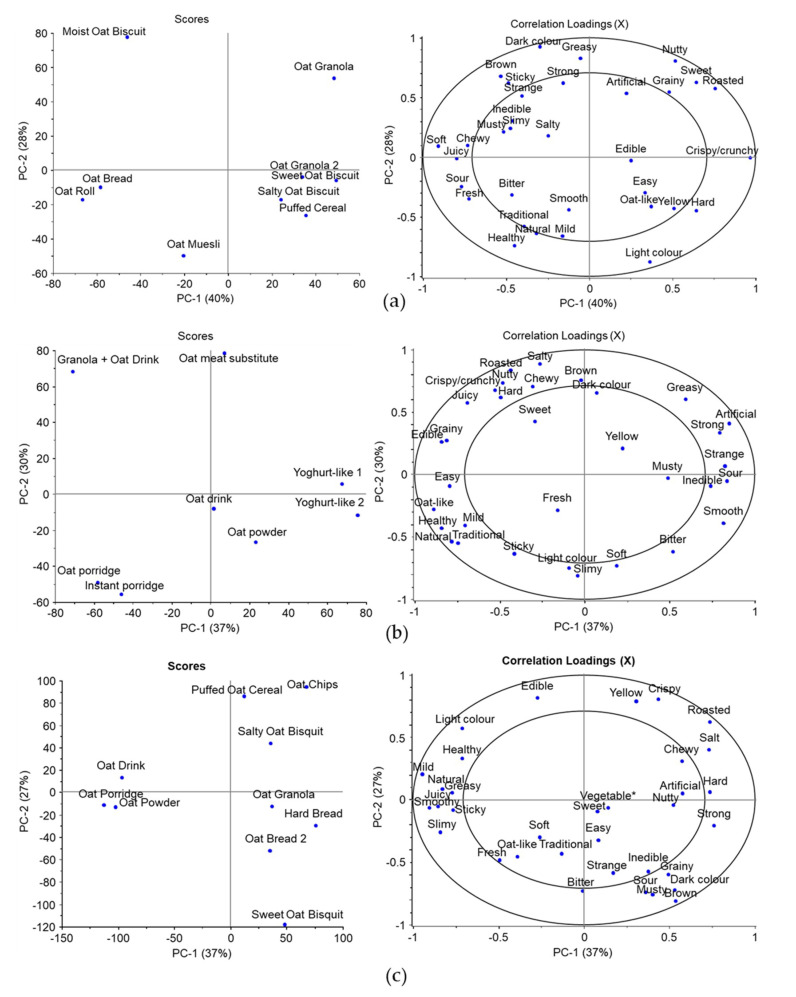
Principal component analysis models based on the Check-All-That-Apply (CATA) attributes used to describe the oat samples in the three sensory tests (Table 1). (**a**) Scores and loadings plots for samples evaluated in study 2; (**b**) samples evaluated in study 3; (**c**) samples evaluated in study 4. Only attributes selected by a minimum of 5% of the panels were used in the models.

The CATA tests were performed in three separate studies (Table 1). In general, the most often checked attributes were “edible”, “light color”, “oat-like”, “mild”, “healthy” and “soft” in the three tests. The three former attributes were the most often selected in both locations and “mild” was among the six most often picked attributes, but there were some differences between panels. In Finland, “healthy” was the fourth most often selected attribute, whereas in Study 4, “healthy” and “soft” were replaced by “chewy” and “roasted”. Three separate PCA models were created using the CATA attribute frequencies (Figure 1). The first two Principal components (PCs) illustrated in Figure 1a–c showed 68%, 67% and 64% of the variances among the datasets. In Figure 1a with the dry oat products (Study 2), some of the most often used descriptors were linked to biscuits and cereal on the right side of the plots (“crispy/crunchy”) and oat breads on the opposite of the first PC (“soft”). On the second PC, “healthy” and “light color” were linked to oat muesli, whereas the moist oat biscuit was described as “strange” and “sticky” and oat granola as “sweet” on the opposite side to healthy but separated by the first PC.

Among the “moist” products included in Study 3 (Figure 1b), the unsweetened oat-based yoghurt-like products were described as “strange”, “sour”, “inedible” and “strong” on the first PC, whereas the oat porridges were more “healthy”, “oat-like” and “natural”. On the second PC and on the opposite side to the porridges was the meat substitute and oat granola + oat drink (a mixture of the two samples evaluated also separately, Table 2) with “chewy”, “salty” and “brown”. The latter mixture sample was also described as “crispy/crunchy” and “roasted”. In Study 4 conducted in China, the porridge and the drinks (milk and powder drink) were described as “natural” and “healthy”, whereas the puffed cereal and oat chips were described as “crispy” and “roasted” on the opposite side of the first PC. The latter two samples were separated from bread samples and sweet biscuit along the second PC with “grainy”, “sour” and “inedible”. The most frequently used attribute, “edible”, located on the opposite side correlated with puffed oat cereal.

**Figure 2:**
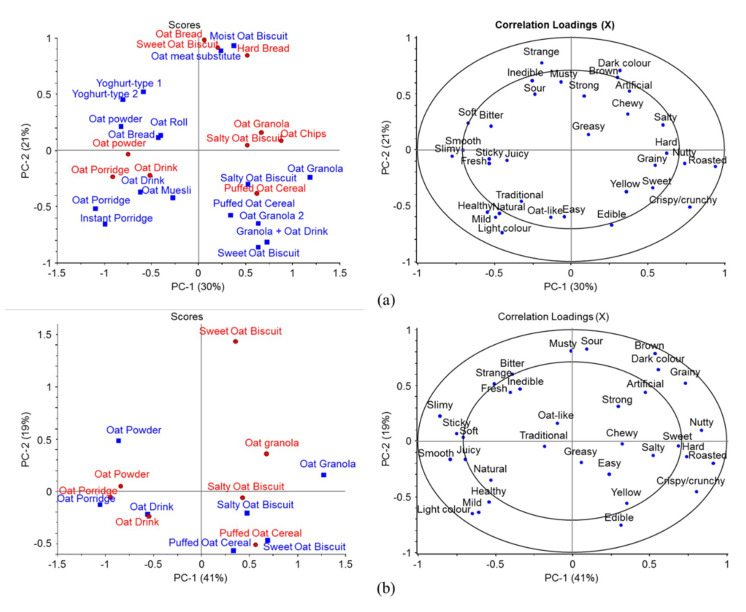
PCA models with (**a**) all samples included in the CATA tests in Finland (blue rectangles) and China (red circles) (*n* = 27) with their sensory descriptors and (**b**) only seven samples included in both locations. Only attributes selected by a minimum of 5% of the panels were used in the models.

In order to compare both test locations, Finland and China, and their descriptors at the same time, additional PCA models were created combining all samples in the tests (*n* = 27) and their sensory descriptors (Figure 2a) and separate model for the seven samples included in both locations (Figure 2b). Three groups of samples were formed on the first two components with 57% of total variance in the first model. On the right on PC-1 in Figure 2a is the group of samples, such as the granolas and puffed cereals and some of the biscuits, described as “crispy/crunchy”, “roasted” and “nutty”, but also “hard”, “sweet”, “grainy” and “edible”. On the opposite side of PC-1 are various moist/wet products, such as the porridges, drinks and yoghurts, with “healthy”, “natural”, “smooth” and “slimy” attributes. Located on the top of PC-2 is the third group of varying samples mostly described as “strange”, “inedible” and “musty” and with “dark” and “brown” colors. The most notable difference between the Finnish and Chinese panels was with the sweet oat biscuit. It was associated with the aforementioned third group in the Chinese test but in the first group, in the Finnish test. Similarly, with the second model (Figure 2b), most of the samples were described similarly in both locations except the sweet oat biscuit along the PC-2.

### 3.3. Comparison of Hedonic Liking Ratings by Finnish and Chinese Participants

Seven of the oat samples shown in Table 2 were included in both test locations. In general, sweeter samples, such as sweet oat biscuit and oat granolas, were the most preferred based on the flavor (Table 4), whereas especially the unsweetened oat powder drink and yoghurt-like products were the most disliked in Finland, and the hard bread, as well as the unsweetened oat powder drink, were the most disliked in China (Table S3). In most cases, the samples were perceived more pleasant by the Finnish in comparison to the Chinese participants. Puffed oat cereal and the oat powder were perceived more pleasant by the Chinese panel. Intensities of oat odor and/or flavor were perceived more intense by the Finnish in sweet biscuit, oat drink and powder drink, and more intense by the Chinese in oat porridge and oat granola.

## 4. Discussion

Currently, there is little published information concerning the sensory properties of oat products as most of studies have focused mainly on the oat itself or on certain specific food processes. Most of the studies have utilized trained panels and descriptive analysis techniques. The benefits of the rapid methods such as the Check-All-That-Apply (CATA) test used in this study are that they can be constructed to include a larger participant number compared to studies with trained panels. The use of CATA in cross-cultural studies is especially reasonable and feasible, as the influence of a single participant is smaller than in trained panels. While CATA methods may be more difficult to interpret than studies with trained panels [33,34], methods focusing on the similarities and differences among products, such as Projective Mapping used in this study, may overcome these issues [22]. In general, the data collection procedures in cross-cultural sensory studies are recommended to be as similar as possible [22]. However, the use of paper forms in Wuxi, for example, instead of computers as in Turku, or the differences in the sample sets in studies 2–4 (Table 2), may have influenced the outcome.

Here, we provided the CATA list in Finnish or English to participants in Finland and in Chinese in study 4 in China. The translations were conducted by a food scientist, a native Chinese speaker with >5 years of experience in living in Finland, and thus with good understanding of food culture in both countries. However, in any cross-cultural study, the possibility remains for words having a slightly different tone in different languages and cultures. Results of the CATA tests were in accordance with literature [12,14] to some extent as “light color” or “mild” were linked to the less processed oat porridges and plain oat muesli and “strong” and “dark color”’ correlated with many of the more processed product samples.

We found ethnicity (country of origin) to have a stronger influence on the clustering of the participants based on the online questionnaire than gender, FNS or GHI status. The participants in cluster 5 consisting largely of non-Finnish responders were less interested or aware of the healthiness of their food compared to the other clusters. The hedonic ratings may also have been influenced by the familiarity of certain foods. Refined and polished grains are traditionally dominant in Chinese food culture, although consumption of whole grain-based products have been an emerging trend with the increasing awareness of the consumers about the health benefits of whole grain products. This is in contrast to the situation in Finland and other Northern European countries, where whole grain-based cereal products have been part of the traditional food culture, and products such as oat breads and different whole grain porridges are well known. Cluster 5 included participants from a wide range of countries, and with varying time spent in Finland prior to participation to the test (data not shown). Thus, some of them may have become more familiar with the local foods and have started using them regularly, whereas many may still prefer to use foods and ingredients familiar to them. Previous studies have indicated that Chinese people who have moved abroad may be more connected to the original Chinese food in comparison to other ethnicities, and the effect may persist in several generations [26]. The fact that cultural differences may have influenced the answering of the questions (e.g., use of scales) should also be recognized [22].

The acceptance of snack-type oat products did not differ between the clusters indicating that they are universally popular. Moreover, among the most important reasons for selecting oat products (Table S2), the Chinese panel in study 4 chose “Easy”. Snack-type foods are also increasing in popularity in Finland, and their consumption may be linked to increasing obesity [35]. Thus, oats have potential as an ingredient in healthier snack-type foods in varying markets.

CATA tests showed similarities and differences between the Finnish and Chinese panelists. As expected, the “sweet” samples, such as granolas and the sweet biscuits, were more preferred in both locations in comparison to the more “sour” or “bitter” unsweetened products (Figure 1 and Figure 2). Interestingly, the sweet biscuit evaluated by the Chinese panel in study 4 was located in the third group of samples (on top of the PC-2 in the scores plot, Figure 2), whereas the corresponding sample evaluated by the Finnish panel was located in the first group on the opposite side of the PC-2. Although being a generally liked sample, it was described differently. Potentially, the descriptor “hard” or characteristics associated with the descriptor “strange” were not familiar to the Chinese panelists. The strangeness can be linked to unfamiliarity and thus decreased liking of the products in the third group. Unfortunately, as a limitation of this study [22], all samples used in studies 2 and 3 in Finland were not studied in China in study 4 due to the required cold storage during transportation. Some of those, e.g., the unsweetened oat yoghurt-like products and the meat substitutes, were among the distinctively described or more disliked samples in study 3. However, the introduction of such products to foreign markets would require costly cooled transportation or local production, and thus is not feasible in comparison to products not requiring cold storage.

Oats are a traditional and important food ingredient in Nordic countries where breads and porridges are part of the traditional daily diet. This is also shown in this study where the breads and porridges were the most familiar product categories in the online questionnaire (Table 3). At the same time, these foods were the most liked. The familiarity of foods plays an important role in liking and in usage of foods [36]. Here, the most novel and thus unfamiliar product categories, such as oat-based meat substitutes, oat powders or oat candies, were more disliked. In China, the dominating ingredients are rice, wheat and soy, and bread is not a traditionally used food item. Thus, oat breads and other oat foods are unfamiliar to Chinese food culture. However, there may be significant regional differences in the preferences. Wheat is grown in the northern parts of China, whereas rice is most popular in the southern regions. Compared to wheat and rice, oat cultivation is limited to few Northern provinces including mainly Shanxi, Shaanxi, Inner Mongolia and Hebei [37]. The oat samples in this study were provided in plastic cups without any additional information about the product (Table 2). For consumers who are familiar to the products, the intrinsic factors of the food play an important role in liking, whereas those consumers who are unfamiliar with them, may rely more on the extrinsic factors, such as information displayed on the product packaging [38]. We chose to present samples “as such” without any additional ingredients where possible. The added ingredients, such as berry flavors in oat yoghurts or added side dishes on top of breads or biscuits, would have affected the perceived familiarity and liking. This would have directed the perspective away from the features of the oat itself towards the added ingredients. Already the sample of granola with added oat milk in study 3 resulted in the highest hedonic ratings among all oat products. This shows the importance of a real life situation and context in conducting the acceptance tests, and calls for further studies in more narrow product categories served in meal type settings. Potentially, oat could be incorporated as an ingredient in local foods or products instead of introducing it in more unfamiliar product forms and concepts. On the other hand, the added ingredients should not undermine or compromise the healthiness of the oat. While it is globally true that the sensory properties of food are the primary driver in food choices of consumers, there are variations between people to the extent of readiness to trade the enjoyment from food for potential health benefits, which was also noted in the results of this study.

The number of new oat-based products has recently skyrocketed in Finland, and separate, sensory tests could be organized using many of the product categories alone. Many of the product categories (Table 3) or actual products (Table 2) are becoming more frequently used and thus more familiar to larger consumer groups. Some consumers may discard all animal-based or wheat-based foods from their diets, while many are ready to test new plant-based alternatives and use them in addition to the traditional options. At the same time, new innovative foods are introduced actively to the markets, some also with oat as the main, or additional yet emphasized ingredient. Especially, the meat or dairy replacers have increased their popularity in recent years. In these product categories, oats have provided a more local option to various other plant-based options, such as soy.

In addition to familiarity, consumer’s food neophobia status has an important role in determining the acceptance of foods, especially with novel and unfamiliar foods [36,39,40,41]. Food neophobia has also been shown to be partially genetically determined [42]. Clusters 1–3 consisted mainly or mostly of less food neophobic participants, whereas clusters 4 and 5 had more neophobic participants (Table 3). In the comparison of clusters 1 and 4, where other factors such as gender, interest in healthiness of food and origin of the participants were considered, the more novel oat drinks were more liked by cluster 1. However, the food neophobia status of the participants in the online questionnaire affected the liking ratings notably less in comparison to participants’ origin. Food neophobia may also affect the stronger flavored foods more than the mild ones [43]. Here, the attribute “mild” was linked to oat porridges (Figure 1 and Figure 2) along with “natural” and “oat-like” attribute indicating the natural “oat” flavor being mild [13,14]. In Figure 2, various food items in the third group, which were more novel for the participants, correlated with the “strong” attribute, and thus, they may have been partially disliked due to food neophobia.

Interestingly, the oat porridges were associated with healthiness in both Finland and China (studies 3 and 4). At the same time, in both locations or in the online questionnaire, the porridges were not among the most or least liked samples or food categories. Potentially, this is due to simplicity and less processing which in some cases may be more liked and often associated with being healthier [44]. However, the liking and the interest to purchase the porridge (Table 4) were rated higher by the Finnish panel. The oat porridge has already been for a long time perceived as a healthy and nutritious option for a breakfast. The healthiness of oats has become increasingly known among the consumers, potentially also in China, especially among the higher educated participants in study 4, i.e., students and staff at the Jiangnan University. As the students may be more open to new food choices and perhaps more aware of the health benefits of oats, they are potential consumers of oat products. Interest to purchase oat products may be more dependent on the consumer’s education level and the nutritive value of the product rather than the product price or the income level of the consumer [45]. Similar to study 4, also in Finland in studies 2 and 3, the participants were mainly highly educated university students and staff, and thus, extrapolation to the whole population should be done with caution [22]. More studies are needed also with more random sampling and participants with a wider socioeconomic background and food preference (deriving from regional differences). Concerning the healthiness of oat, the GHI status of the participants affected mainly the porridge category in the online questionnaire as the more health interested in cluster 1 gave higher ratings compared to the corresponding cluster 2 with lower health interest. As the healthiness of oat may be a potential marketing strategy for various product categories, familiarity and sensory properties likely have stronger impacts on the purchasing decision by consumers who do not emphasize the healthiness of foods.

In addition to being associated with healthiness, porridges were associated with the “natural” attribute in Figure 1 and Figure 2. The naturalness of foods is a very important factor in food choices for most of the consumers and it can be classified by its origin, technologies and ingredients used or by the characteristics of the final product [46]. Here, the porridges in studies 3 and 4, as well as the plain muesli in study 2, were potentially perceived as less processed and with lesser ingredients in comparison to the other samples. Moreover, the oat-like odor and flavor perceived from the porridges were the most intense among the samples (Table 4). Some of the products included in this study, and also many oat products currently in market, may contain relatively little oat in comparison to the other ingredients, thus resulting in the lower intensity of oat-like odors and flavors.

In conclusion, the acceptance of oat products and product categories was studied in Finland and China. The ethnicity of the participants had an important role affecting the rated pleasantness and familiarity of oat product categories, whereas both food neophobia and health interest status also had an influence. The effects of these were observed in different oat product categories. For example, the ethnicity affected the acceptance of the traditional and generally liked foods, such as breads and food neophobia status, and the acceptance of novel and familiar foods, such as meat substitutes. The health interest status affected the acceptance of oat porridges, which were also universally described as “healthy”. The sensory tests using the CATA method showed several similarities between the Finnish and Chinese panel in the selection of sensory descriptors for product. In addition to porridge being generally described as “healthy”’, oat granolas were described as “sweet”’ and “roasted”’ in both locations. The generally liked sweet oat biscuits were described differently in two tests.

Based on this study, different strategies are needed for different oat products in order to introduce them successfully to consumers in Finland, where the oat is a significant part of consumers’ daily diets, or China, where oats are only regionally known or only becoming more popular. However, the healthiness of the oat is a crucial factor affecting the choices of consumers in both countries, thus strongly affecting the acceptance of “oat-like” products, such as porridges. Sweetness is also a generally liked attribute among the products in this study, but too much added sugar results in reducing the potential healthiness of oats. However, more studies are needed focusing on the consumer acceptance and usage of oats and oat products, especially in different varying consumer segments, in order enhance their consumption. The study provides assistance to food companies in bringing products to new markets or to new consumer groups. This applies both to marketing oat products in Western countries, where the oat is traditionally consumed, and to exporting oat products to markets where the oat is not yet equally well utilized and accepted. The study also benefits researchers studying multicultural differences especially in relation to preference for grains or foods in general.

## Figures and Tables

**Table 1:** Characteristics of the four studies conducted.

	Study 1	Study 2	Study 3	Study 4
Location	Finland	Turku, Finland	Turku, Finland	Wuxi, China
Time of data collection	April–June 2017	November 2017, January 2018	November–December 2017, January 2018	June 2018
Test method	Online questionnaire	Sensory evaluation in laboratory	Sensory evaluation in laboratory	Sensory evaluation in laboratory
Number of sample categories or samples	11	9	8	10
Number of participants	381	65	73	103
Females, %	75.9	70.8	60.3	81.6
Age range (median)	18−76 (29)	18−68 (27)	18−68 (27)	18−36 (24)
Country of origin, %				
Finland	83.7	72.3	68.5	-
China	7.6	20.0	19.2	100
Other	8.7	7.7	12.3	-

**Table 2:** Oat samples, their preparations, served amounts and serving conditions in the sensory tests in Finland and China.

Sample	Study	Preparation	Served Amount	Serving Dish	Serving Temperature
Sweet Oat Biscuit	2, 4	Split piece into halves	Two halves	Glass cup	Room temp
Salty Oat Biscuit *	2, 4	as such	Two biscuits	Glass cup	Room temp
Puffed Oat Cereal	2, 4	as such	2 tablespoons	Glass cup	Room temp
Oat Drink	3, 4	Shaken	20 mL	Plastic cup	Refrigerated
Oat Powder	3, 4	1 part oat powder + 4 parts water, mixed until homogenized	20 mL	Plastic cup	Refrigerated
Oat Porridge	3, 4	450 mL water mixed with 200 mL flakes, gently simmered over low heat for 15 min in Finland, 3 min in microwave (full power) in China	1 tablespoon	Glass cup	Warm (60 °C)
Oat Granola **	2, 4	as such	2 tablespoons	Glass cup	Room temp
Oat Bread	2	Defrosted (−18 °C) in microwave oven, edges cut away, cut into 6 pieces	One rectangular piece (approx. 2 × 3 cm)	Glass cup	Room temp
Moist Oat Biscuit	2	as such	One piece	Glass cup	Room temp
Oat Roll	2	Defrosted (−18 °C) in microwave oven, edges cut away, cut into 4 pieces	One square piece (approx. 3 × 3 cm)	Glass cup	Room temp
Oat Granola 2 *	2	as such	2 tablespoons	Glass cup	Room temp
Oat Muesli *	2	as such	2 tablespoons	Glass cup	Room temp
Yoghurt-alternative 1	3	Stirred	2 teaspoons	Plastic cup	Refrigerated
Yoghurt-alternative 2	3	Stirred	2 teaspoons	Plastic cup	Refrigerated
Instant Porridge	3	250 mL hot water from electric kettle mixed with 150 mL flakes, simmered for 5 min	1 tablespoon	Glass cup	Warm (60 °C)
Oat Meat Substitute	3	Heated in microwave oven (full power) for 30 s	1 tablespoon	Glass cup	Warm (60 °C)
Granola in Drink *	3	Oat drink shaken	1 tablespoon granola + 15 mL oat drink	Glass cup	Refrigerated
Oat Chips	4	as such	One piece	Plastic cup	Room temp
Hard Bread	4	Split piece in halves	One half	Plastic cup	Room temp
Oat Bread 2	4	Defrosted (−18 °C) in microwave oven, cut into 6 pieces	One rectangular piece (approx. 2 × 3 cm)	Plastic cup	Room temp

* Modified versions of the commercial products without added flavoring ingredients provided by the manufacturer. Granola in drink was mixed by the participant right before testing the sample. ** Manufactured in Sweden. All other products were manufactured in Finland.

**Table 3:** Comparison of participant clusters ^a^ and the rated liking of oat product categories in the online questionnaire.

	Cluster 1	Cluster 2	Cluster 3	Cluster 4	Cluster 5	All
Number of participants	111	77	69	62	62	381
Gender (%) ^a^	Female (100)	Female (100)	Male (91.3)	Female (100)	Female (61.3)	
Country of origin (%) ^a^	Finland (100)	Finland (100)	Finland (100)	Finland (100)	Other (100)	
Food Neophobia Scale ^a^	Less FN	Less FN	Less FN	More FN	More FN	
Less neophobic %	100	57.1	65.2	45.2	0	59.8 (21.2)
More neophobic %	0	42.9	34.8	54.8	100	40.2 (38.0)
General Health Interest ^a^	More HI	Less HI	Less HI	More HI	More HI	
Less interested %	0	100	53.6	33.9	0	35.4 (28.4)
More interested %	100	0	46.4	66.1	100	64.6 (43.3)
Interest in healthiness of food ^b^	6.23 ± 0.74 a	5.14 ± 0.90 c	5.42 ± 1.10 bc	6.26 ± 0.72 a	5.58 ± 1.35 b	5.76 ± 1.06
Awareness of healthiness of food ^b^	5.93 ± 0.93 a	5.55 ± 0.95 ab	5.57 ± 0.93 ab	5.97 ± 0.77 a	5.32 ± 1.44 b	5.69 ± 1.04
Usage of products containing oats ^b^	2.27 ± 1.41 b	2.56 ± 1.29 b	2.75 ± 1.46 b	2.63 ± 1.36 b	3.47 ± 1.80 a	2.67 ± 1.50
Pleasantness rating of oat product categories ^b^						
Oat breads	8.32 ± 0.87 a	8.01 ± 1.16 ab	7.54 ± 1.38 b	8.10 ± 1.14 ab	6.34 ± 1.99 c	7.76 ± 1.46 A
Oat porridges	8.05 ± 1.17 a	7.10 ± 1.89 bc	6.83 ± 1.86 cd	7.82 ± 1.68 ab	6.10 ± 2.35 d	7.28 ± 1.89 B
Oat mueslis	6.94 ± 1.60 ab	6.43 ± 1.63 ab	6.38 ± 1.76 ab	6.97 ± 1.76 a	6.11 ± 2.42 b	6.60 ± 1.84 CD
Oat flakes and brans	6.94 ± 1.79 a	6.66 ± 1.66 a	5.83 ± 1.79 b	6.85 ± 1.45 a	5.56 ± 1.78 b	6.44 ± 1.79 DE
Oat powders	4.90 ± 1.73	4.81 ± 1.56	4.42 ± 1.58	5.15 ± 1.77	4.71 ± 1.81	4.80 ± 1.70 G
Oat meat substitutes	6.52 ± 1.83 a	6.03 ± 1.75 a	5.71 ± 2.16 a	6.15 ± 2.09 a	4.77 ± 2.16 b	5.93 ± 2.05 F
Snack-type oat biscuits	6.66 ± 1.71	6.95 ± 1.54	6.93 ± 1.52	6.77 ± 1.64	6.79 ± 1.58	6.81 ± 1.61 CD
Coffee-table oat biscuits	6.80 ± 1.87 a	7.26 ± 1.66 a	7.07 ± 1.73 a	6.90 ± 1.46 a	6.37 ± 2.06 b	6.89 ± 1.79 BC
Oat-based yoghurts	6.69 ± 1.91 a	6.08 ± 2.08 ab	5.42 ± 1.97 b	5.71 ± 2.01 ab	6.18 ± 2.28 b	6.09 ± 2.08 EF
Oat drinks	6.57 ± 2.05 a	5.61 ± 2.27 ab	4.96 ± 2.20 b	5.42 ± 2.21 b	5.40 ± 2.28 b	5.71 ± 2.25 F
Oat candies	4.80 ± 1.75	4.66 ± 1.77	4.35 ± 1.96	4.56 ± 1.71	4.97 ± 2.19	4.68 ± 1.86 G
Familiarity rating of oat product categories ^b^						
Oat breads	4.41 ± 0.59 a	4.25 ± 0.67 a	4.23 ± 0.77 a	4.26 ± 0.68 a	3.48 ± 1.18 b	4.17 ± 0.83 A
Oat porridges	4.71 ± 0.49 a	4.36 ± 0.72 ab	4.29 ± 0.73 b	4.56 ± 0.64 ab	3.81 ± 1.28 c	4.39 ± 0.83 A
Oat mueslis	3.49 ± 0.97	3.21 ± 1.03	3.42 ± 0.93	3.47 ± 1.02	3.19 ± 1.45	3.37 ± 1.08 CD
Oat flakes and brans	3.87 ± 0.95 a	3.45 ± 1.08 ab	3.38 ± 0.81 b	3.58 ± 0.76 ab	2.73 ± 1.31 c	3.46 ± 1.06 BC
Oat powders	2.07 ± 0.97 a	1.48 ± 0.74 b	1.81 ± 1.03 ab	2.08 ± 1.16 a	2.11 ± 1.01 a	1.91 ± 1.01 F
Oat meat substitutes	3.37 ± 0.96 a	2.78 ± 1.07 b	3.03 ± 1.01 ab	3.03 ± 1.06 ab	2.27 ± 1.28 c	2.96 ± 1.12 E
Snack-type oat biscuits	3.64 ± 0.71	3.58 ± 0.66	3.70 ± 0.71	3.60 ± 0.84	3.56 ± 0.88	3.62 ± 0.75 B
Coffee-table oat biscuits	3.56 ± 0.66 a	3.68 ± 0.52 a	3.77 ± 0.71 a	3.48 ± 0.76 a	3.06 ± 1.21 b	3.53 ± 0.81 BC
Oat-based yoghurts	3.48 ± 1.09 a	3.29 ± 0.99 ab	2.93 ± 0.94 b	2.98 ± 0.88 b	3.03 ± 1.29 ab	3.19 ± 1.07 D
Oat drinks	3.63 ± 1.12 a	3.12 ± 1.21 ab	2.93 ± 1.10 b	3.06 ± 1.08 b	2.77 ± 1.21 b	3.17 ± 1.18 DE
Oat candies	1.49 ± 0.84 ab	1.27 ± 0.70 b	1.49 ± 0.85 ab	1.35 ± 0.63 b	1.77 ± 1.09 a	1.47 ± 0.84 G

^a^ Clusters based on four class variables: gender and country of origin (Table 1), Food Neophobia Scale (FNS, [31]) status and General Health Interest (GHI, [32]) status. FNS and GHI scores of all 381 participants were first used to create two subsets (less FN/HI or more FN/HI). Mean FNS or GHI scores for the two subsets among all participants are shown brackets after percentages. ^b^ Mean ratings (± standard deviations; scale 1–7; 1 = not interested/aware/familiar, 7 = extremely interested/aware/familiar; scale for usage: 1 = 2–4 times a day, 2 = Once a day, 3 = 2–4 times a week, 4 = Once a week, 5 = 1–3 times a month, 6 = A few times a year, 7 = Never; hedonic liking ratings on a balanced scale 1–9) for clusters and all participants. Statistical differences (if found) are based on ANOVA with Tukey’s post hoc test (*p* < 0.05) and are shown with lowercase letters a–d between clusters and uppercase letters A–G between samples in general.

**Table 4:** Comparison of the rated pleasantness, intensities oat odor and flavor and interest to purchase for the oat samples.

Samples ^a^ Participants		Pleasantness ^b^				Intensity ^b^		Purchase Interest ^b^
		Appearance	Odor	Flavor	MF & T	Oat Odor	Oat Flavor	
Sweet Oat Biscuit	China (*n* = 103)	6.30 ± 1.2	6.56 ± 1.2	7.17 ± 1.4 b	6.82 ± 1.2 b	3.97 ± 1.4 b	4.24 ± 1.5 b	3.65 ± 1.1 b
	Finland (*n* = 65)	6.43 ± 1.6	6.38 ± 1.5	7.60 ± 1.6a	7.30 ± 1.3 a	4.46 ± 1.6 a	4.74 ± 1.5 a	4.03 ± 1.0 a
Salty Oat Biscuit	China (*n* = 103)	5.45 ± 1.3	5.33 ± 1.1	5.64 ± 1.5	5.67 ± 1.5 b	3.52 ± 1.4	3.99 ± 1.5	2.95 ± 1.1
	Finland (*n* = 65)	5.32 ± 1.5	5.65 ± 1.5	5.91 ± 1.8	6.35 ± 1.6 a	3.62 ± 1.6	4.15 ± 1.4	2.69 ± 1.3
Puffed Oat Cereal	China (*n* = 103)	6.50 ± 1.4 a	5.84 ± 1.1 a	6.77 ± 1.2 a	6.93 ± 1.3	2.57 ± 1.4	2.94 ± 1.3	3.44 ± 1.1
	Finland (*n* = 65)	5.79 ± 1.9 b	4.95 ± 1.6 b	6.29 ± 1.7 b	7.03 ± 1.5	2.37 ± 1.4	3.05 ± 1.7	3.06 ± 1.5
Oat Drink	China (*n* = 103)	5.90 ± 1.2	5.28 ± 0.8 b	6.20 ± 1.4 b	6.33 ± 1.6	1.95 ± 1.1	2.73 ± 1.5 b	3.07 ± 1.2
	Finland (*n* = 73)	5.97 ± 1.9	5.60 ± 1.0 a	6.60 ± 1.6 a	6.82 ± 1.8	1.79 ± 1.2	3.49 ± 1.7a	3.38 ± 1.4
Oat Powder	China (*n* = 103)	5.51 ± 1.2	5.00 ± 0.8	4.20 ± 1.7 a	4.52 ± 1.8 a	2.70 ± 1.6 b	3.51 ± 1.8 b	2.08 ± 1.1 a
	Finland (*n* = 73)	5.15 ± 1.6	5.05 ± 1.6	3.32 ± 1.7 b	3.70 ± 1.9 b	3.63 ± 1.9 a	4.51 ± 1.6 a	1.48 ± 0.8 b
Oat Porridge	China (*n* = 103)	4.58 ± 1.7 b	5.53 ± 1.6 b	5.19 ± 1.6 b	5.13 ± 1.8 b	4.90 ± 1.6 a	5.13 ± 1.7 a	2.50 ± 1.2 b
	Finland (*n* = 73)	6.05 ± 1.7 a	6.62 ± 1.3 a	6.33 ± 1.4 a	6.63 ± 1.4 a	4.27 ± 1.5 b	4.36 ± 1.7 b	3.78 ± 1.2 a
Oat Granola	China (*n* = 103)	6.30 ± 1.4 b	6.25 ± 1.3 a	6.83 ± 1.5 b	6.26 ± 1.4 b	3.77 ± 1.6	4.73 ± 1.5 a	3.56 ± 1.1
	Finland (*n* = 73)	7.09 ± 1.6 a	5.66 ± 1.8 b	7.77 ± 1.1 a	7.63 ± 1.2 a	3.51 ± 1.8	4.06 ± 1.6 b	3.82 ± 1.3

^a^ Samples included both in Finland and China. ^b^ The evaluation scale was a balanced hedonic 9-point scale for the pleasantness (MF & T mouthfeel and texture), 7-point scale for the intensities and 5-point scale for the purchase interest. Rated attributes presented to panelists as in the table after completion of the Check-All-That-Apply (CATA) test. Sample information presented in Table 2. The letters a–b marked the significant statistical differences (*p* < 0.05) within each comparison.

## Supplementary Materials

The following are available online at https://www.mdpi.com/2304-8158/9/9/1234/s1, Table S1: List of Check-All-That-Apply (CATA) attributes for describing the samples as presented to panelists in Finland (English and Finnish) and in China (Chinese). Table S2: The most important reasons ^a^ for selecting oat products presented as percentages of the participants. Table S3: Mean hedonic ratings and standard deviations (on a scale 1–9) for samples included in the sensory tests.
