# Supplementary material for: Sensory Characteristics Contributing to Pleasantness of Oat Product Concepts by Finnish and Chinese Consumers
Source: Foods. 2020 Sep 4;9(9):1234. doi: 10.3390/foods9091234 (PMC7556016; doi:10.3390/foods9091234)
Supplement: Supplementary file 1 [file foods-09-01234-s001.docx]

**Supplementary Materials of Sensory Characteristics Contributing to Pleasantness of Oat Product Concepts by Finnish and Chinese Consumers**

**Authors: Oskar Laaksonen, Xueying Ma, Eerika Pasanen, Peng Zhou, Baoru Yang and Kaisa M. Linderborg**

Table S1: List of Check-All-That-Apply (CATA) attributes for describing the samples as presented to panelists in Finland (English and Finnish) and in China (Chinese).

| Descriptors (in the order of presented to panelists) | |
| --- | --- |
|  Oat-like / kauramainen / 像燕麦 |  Slimy / limainen / 粘糊糊的 |
|  Salty / suolainen / 咸 |  Roasted / paahteinen / 烘烤的 |
|  Sweet / makea / 甜 |  Musty / tunkkainen / 霉味的 |
|  Sour / hapan / 酸 |  Fresh / tuore, raikas / 新鲜的 |
|  Bitter / karvas / 苦 |  Healthy / terveellinen / 健康的 |
|  Strong / vahva / 强烈的 |  Natural / luonnollinen / 天然的 |
|  Mild / mieto / 温和的 |  Artificial / keinotekoinen / 人工的 |
|  Hard / kova / 硬 |  Traditional / perinteinen / 传统的 |
|  Soft / pehmeä / 软 |  Easy / helppo / 容易的 |
|  Crispy, crunchy / rapea / 脆 |  Strange / outo / 奇怪的 |
|  Chewy / sitkeä / 咀嚼感 |  Edible / syömäkelpoinen / 可食用的 |
|  Juicy / mehevä / 多汁的 |  Inedible / syömäkelvoton / 非食用的 |
|  Grainy / rakeinen / 粒状的 |  Light colour / vaalea / 浅色的 |
|  Smooth / sileä / 细腻顺滑的 |  Dark colour / tumma / 深色的 |
|  Sticky / tahmea / 粘 |  Yellow / keltainen / 黄色的 |
|  Greasy / rasvainen / 腻 |  Brown / ruskea / 棕色的 |
|  Nutty / pähkinäinen / 坚果般的 |  (Other) / 其他 * |

Table S2: The most important reasons^a^ for selecting oat products presented as percentages of the participants.

|  | Online questionnaire (study 1) | | | China (study 4) |
| --- | --- | --- | --- | --- |
|  | All | Finland | Other country |  |
|  | (n=381) | (n=319) | (n=62) | (n=103) |
| Flavor | **70.3** | **76.8** | 37.1 | **70.9** |
| Rich in fiber | **67.7** | **67.7** | **67.7** | - |
| Healthy | **67.5** | **66.8** | **71.0** | **63.1** |
| Wellbeing of stomach | 52.0 | 54.5 | 38.7 | - |
| Easy | 43.0 | 43.9 | 38.7 | **60.2** |
| As an alternative | 37.8 | 39.2 | 30.6 | - |
| Price | 27.3 | 30.1 | 12.9 | 41.7 |
| Traditional | 27.0 | 30.4 | 9.7 | - |
| Betaglucan lowers blood cholesterol | 24.1 | 25.1 | 19.4 | - |
| Variation of product types | 20.2 | 20.7 | 17.7 | - |
| Low glycemic index | 17.8 | 17.6 | 19.4 | - |
| Vegetarian | 17.6 | 20.1 | 4.8 | - |
| Several flavor alternatives | 12.9 | 12.2 | 16.1 | - |
| Other | 6.8 | 7.8 | 1.6 | 45.6 |
| Trendy food | 5.2 | 5.0 | 6.5 | - |
| Gluten-free diet | 5.0 | 4.7 | 6.5 | - |
| I don't know | 2.4 | 1.9 | 4.8 | - |
| I do not use | 0.5 | 0.3 | 1.6 | - |

^a^ Participants instructed to pick 1–3 most important reasons. The list of reasons differed in China thus the less frequent picks are combined to Other. The most frequent picks highlighted with bold font.

Table S3. Mean hedonic ratings and standard deviations (on a scale 1–9) for samples included in the sensory tests.

| **Sample** | **Appearance** | **Odor** | **Mouthfeel & texture** | **Flavor** |
| --- | --- | --- | --- | --- |
| **Study 2 (Finland; n=65)** | | | | |
| Oat granola 1 | 7.1 ± 1.6 | 5.7 ± 1.8 | 7.6 ± 1.2 | 7.8 ± 1.1 |
| Sweet oat biscuit | 6.4 ± 1.6 | 6.4 ± 1.5 | 7.3 ± 1.3 | 7.6 ± 1.2 |
| Oat granola 2 | 6.7 ± 1.4 | 6.5 ± 1.5 | 6.6 ± 1.2 | 6.8 ± 1.3 |
| Oat Roll | 7.7 ± 1.3 | 7.1 ± 1.6 | 7.0 ± 1.5 | 6.7 ± 1.6 |
| Puffed oat cereal | 5.8 ± 1.9 | 4.9 ± 1.6 | 7.0 ± 1.5 | 6.3 ± 1.7 |
| Oat bread 1 | 7.5 ± 1.2 | 5.3 ± 1.8 | 6.4 ± 1.7 | 6.1 ± 1.7 |
| Salty oat biscuit | 5.3 ± 1.5 | 5.6 ± 1.5 | 6.4 ± 1.6 | 5.9 ± 1.8 |
| Moist oat biscuit | 5.4 ± 1.9 | 6.5 ± 2.0 | 5.0 ± 1.9 | 5.1 ± 2.1 |
| Oat muesli | 6.4 ± 1.6 | 5.5 ± 1.1 | 3.9 ± 1.7 | 4.7 ± 1.9 |
| **Study 3 (Finland; n=73)** | | | | |
| Granola + oat drink | 5.8 ± 1.8 | 7.1 ± 1.3 | 7.5 ± 1.3 | 7.8 ± 1.0 |
| Oat drink | 6.0 ± 1.9 | 5.6 ± 1.0 | 6.8 ± 1.6 | 6.6 ± 1.8 |
| Oat porridge | 6.1 ± 1.7 | 6.6 ± 1.3 | 6.6 ± 1.4 | 6.3 ± 1.4 |
| Instant oat porridge | 6.1 ± 1.6 | 6.6 ± 1.4 | 5.7 ± 1.7 | 5.9 ± 1.6 |
| Oat meat substitute | 4.8 ± 2.1 | 4.3 ± 1.8 | 6.0 ± 2.1 | 5.7 ± 2.0 |
| Oat yoghurt | 5.8 ± 1.9 | 6.0 ± 1.8 | 6.6 ± 1.5 | 4.8 ± 2.1 |
| Oat yoghurt 2 | 6.6 ± 1.5 | 5.8 ± 1.8 | 6.7 ± 1.6 | 3.9 ± 2.1 |
| Oat powder drink | 5.2 ± 1.6 | 5.1 ± 1.6 | 3.7 ± 1.9 | 3.3 ± 1.7 |
| **Study 4 (China; n=103)** | | | | |
| Sweet biscuit | 6.3 ± 1.2 | 6.6 ± 1.2 | 6.8 ± 1.4 | 7.2 ± 1.3 |
| Oat granola 1 | 6.3 ± 1.4 | 6.3 ± 1.3 | 6.3 ± 1.6 | 6.8 ± 1.4 |
| Puffed oat cereal | 6.5 ± 1.4 | 5.8 ± 1.1 | 6.9 ± 1.3 | 6.8 ± 1.2 |
| Oat drink | 5.9 ± 1.2 | 5.3 ± 0.8 | 6.3 ± 1.4 | 6.2 ± 1.6 |
| Oat chips | 6.1 ± 1.3 | 5.7 ± 1.5 | 6.1 ± 1.5 | 5.9 ± 1.6 |
| Salty oat biscuit | 5.5 ± 1.3 | 5.3 ± 1.1 | 5.7 ± 1.5 | 5.6 ± 1.5 |
| Oat porridge | 4.6 ± 1.7 | 5.5 ± 1.6 | 5.1 ± 1.8 | 5.2 ± 1.6 |
| Oat bread 2 | 4.6 ± 1.6 | 3.8 ± 1.7 | 4.7 ± 1.8 | 4.3 ± 1.6 |
| Oat powder drink | 5.5 ± 1.2 | 5.0 ± 0.8 | 4.5 ± 1.8 | 4.2 ± 1.7 |
| Hard bread | 4.6 ± 1.2 | 4.8 ± 0.9 | 3.1 ± 1.5 | 3.5 ± 1.3 |
